# Statin Induced Autoimmune Necrotizing Myopathy (SIANM): An Alarming Adverse Event of a Familiar Medication

**DOI:** 10.7759/cureus.22273

**Published:** 2022-02-16

**Authors:** Nagapratap Ganta, Dina Alnabwani, Veera Jayasree L Bommu, Sharon Hechter, Viraj Shah, Pramil Cheriyath

**Affiliations:** 1 Internal Medicine, Hackensack Meridian Ocean Medical Center, Brick, USA; 2 Internal Medicine, Rajarshee Chhatrapati Shahu Maharaj Government Medical College, Kolhapur, IND

**Keywords:** anti-hmg co-a reductase antibodies, immune-mediated necrotizing myopathy, hypercholesterolemia, statin-induced myopathy, hyperlipidemia, sianm, statin-induced autoimmune necrotizing myopathy

## Abstract

Statins are a widely prescribed medication that lowers serum cholesterol by inhibiting the HMG-CoA reductase enzyme, a rate-limiting step in cholesterol synthesis. Myopathy is one of the well-known adverse effects of statins, mainly when prescribed with the fibrates. However, statin-induced autoimmune necrotizing myopathy (SIANM) is an infrequent and severe complication. Hence, all clinicians should be more vigilant regarding this complication and treat it early to prevent acute kidney injury (AKI).

## Introduction

Statins are a class of drugs commonly used to prevent cardiovascular events by lowering serum total cholesterol and triglyceride levels [1]. According to cohort studies supported by randomized trials, the incidence of myopathy in patients treated with statins was 11 per 100,000 person-years [2]. While self-limited statin myopathy is widely prevalent, statin-induced autoimmune necrotizing myopathy (SIANM) is extremely rare and more severe, with an annual incidence of about two cases per million [3]. We report a rare case of statin-induced autoimmune necrotizing myopathy in a 67-year-old male patient.

## Case presentation

A 67-year-old male with a past medical history of hypercholesterolemia, type II diabetes mellitus, hypertension, and liver disease presented to the emergency department (ED) for outpatient lab abnormalities concerning transaminitis (aspartate aminotransferase (AST)/ alanine aminotransferase (ALT) of 274/198). He reported severe proximal weakness in his thighs and shoulders that started insidiously and progressively became worse over several months. He also endorsed intermittent difficulty with swallowing. In addition, he also reported having multiple recent falls from which he was usually able to get up himself except for the most recent one; however, he was never on the ground for more than an hour at most. In addition, he was found to have some right upper quadrant (RUQ) tenderness for which a RUQ ultrasound was obtained which was unremarkable except for an indeterminate echogenic lesion that was followed up with a magnetic resonance imaging (MRI) of the abdomen with and without contrast which did not show any concerning findings. The patient had no known allergies. He reported that he has been smoking cigarettes, about 0.50 packs per day, and alcohol use but denied the use of drugs. His family history was non-contributory. A review of the other systems revealed no abnormalities.

On examination, he was alert and oriented with dry mucous membranes. Vitals were stable with blood pressure (BP) of 113/56 mmHg, heart rate (HR) of 68 bpm, and oxygen saturation (SpO2) of 96%. Acetaminophen 650 mg and 1000mL of intravenous normal saline bolus were administered. His complete blood picture (CBC), renal function tests, basic metabolic panel (BMP), other liver function tests, ammonia, amylase, lipase, troponin were normal, reverse transcription polymerase chain reaction (RT-PCR) test for SARS-COV-2 came out negative. 

Creatinine phosphokinase (CPK) level was checked due to fall history and was found to be elevated at 21,800 IU/L (22-232 IU/L) concerning rhabdomyolysis. His creatine kinase myocardial band (CK-MB) was >600 IU/L (5-25 IU/L), lactate dehydrogenase (LDH) was 2478 U/L (140-280 U/L), and myoglobin was 4329 ng/mL (25-72 ng/mL). Due to the patient not having any physical markers of a traumatic injury or fall with a long time on the ground, his statin medication was empirically discontinued because it may be statin-induced myopathy. He was treated with intravenous fluids. The CPK was down-trending until it reached a plateau and then started going up, at which point autoimmune myositis was suspected, and a thorough rheumatologic workup was performed.

**Figure 1:**
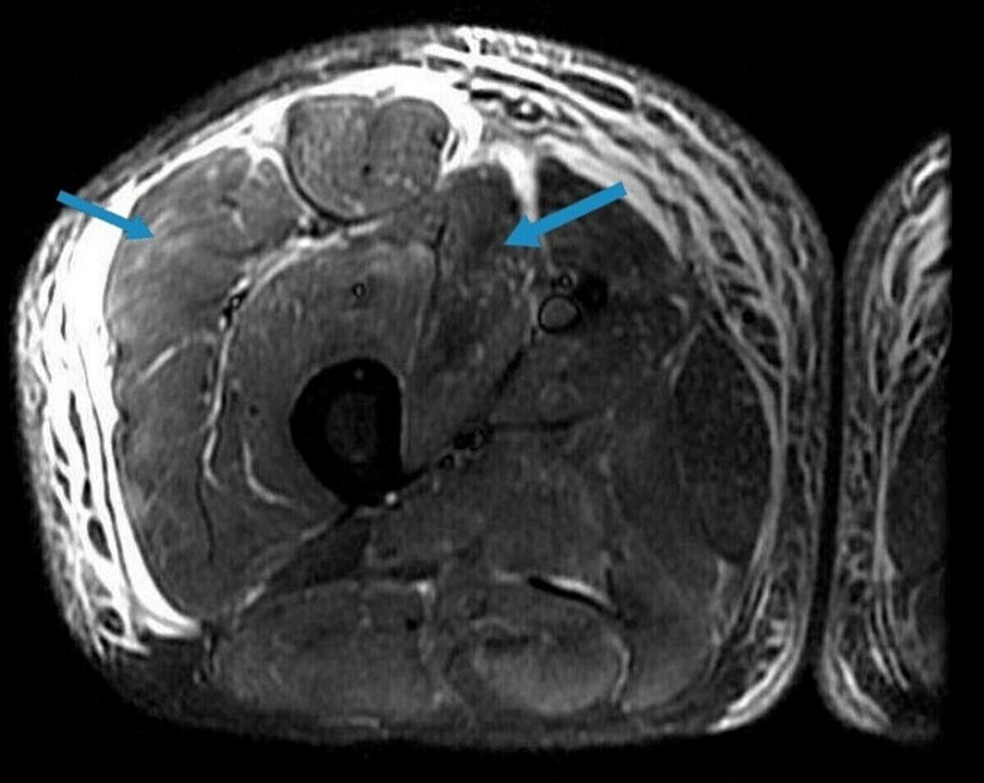
MRI of right thigh showing inflammatory changes (blue arrows) suggestive of myositis

The patient had a negative myositis panel and positive anti-3-hydroxy-3-methylglutaryl-coenzyme-A reductase (HMGCR) IgG antibody concerning statin-induced autoimmune myopathy. The patient received an MRI of the right thigh with and without contrast which showed inflammatory changes suggestive of myositis (Figure 1). MRI-guided biopsy of the right thigh was performed, and he was started on oral prednisone 60 mg once daily, which decreased the CPK levels. Muscle biopsy revealed an inflammatory type of immune-mediated necrotizing myopathy (IMNM) with coexistent neurogenic atrophy. On the day of the discharge, the patient's CPK level was 3,208 IU/L with improvement in his clinical symptoms as well as lab results compared to the day of admission. He was advised to avoid statins in the future. Alternative treatment options such as intravenous immunoglobulins (IVIG), methotrexate, and rituximab were also considered. However, the patient declined IVIG due to his cultural beliefs, and methotrexate was not an option for him due to his transaminitis. He was discharged to a rehabilitation facility on prednisone 20 mg thrice daily. Rituximab was considered for him, and he was advised to follow up with the rheumatologist regarding the same. The patient will be monitored on a long-term basis to comment on the effectiveness of the current treatment plan.

## Discussion

Statins inhibit the HMG-CoA reductase enzyme, a rate-limiting step of cholesterol synthesis. Statins are known to be protective against atherogenesis and lower mortality from cardiovascular events [1]. Creatine kinase (CK) is an enzyme generated by injured muscle cells, and CK elevations >10 times the upper limit of normal (ULN) occur in one to 10,000 people every year. The incidence of adverse effects with lovastatin, simvastatin, or atorvastatin was higher (4.2 per 100,000 person-years) as they are oxidized by cytochrome P450 3A4 (CYP3A4, which is blocked by several medicines) than pravastatin or fluvastatin (which are not oxidized by CYP3A4) [2]. Necrotizing autoimmune myopathy (NAM) is one of the most common inflammatory myopathies, accounting for 19% of all inflammatory myopathies [3].

SIANM's pathophysiological mechanism is not well understood. There is some evidence that statin muscle side effects are more likely in patients with predisposing characteristics such as elevated CPK levels, a family history of myopathy, or a previous diagnosis of neuromuscular disorders or hypothyroidism. Furthermore, some hereditary variables may enhance the likelihood of statin-induced muscle damage [4]. A substantial link between SIANM and HLA-DR11 as well as lower levels of HLA-DQA1 and HLA-DQB6 alleles and HMGCR antibodies was found through genetic association studies. These findings were noted in both Native Americans and African Americans [5]. African American individuals, in particular, have a higher CPK level in their blood and are less sensitive to treatment [6]. The formation of anti-HMG-CoA reductase autoantibodies has been linked to the class II HLA allele DRB1 11:01. In these genetically vulnerable people, statin exposure is thought to cause overexpression of HMGCR antibodies in regenerated myofibers. HMGCR and signal recognition particle (SRP) proteins, as well as classical pathway activation of complement proteins, were detected in the sarcolemma of muscle fibers in affected patients [7]. However, it is not studied whether this increased expression of HMGCR antibodies can be found without statin exposure. The necessity for immunosuppressive therapy, lack of improvement after stopping statins, and frequent relapse when treatment has decreased point to an immune-mediated etiology for this unusual SIANM [8].

Our patient had a severe generalized weakness (his reason for ED visit), creatinine phosphokinase (CPK) was elevated at 21,800 IU/L (22-232 IU/L), CK-MB was >600 IU/L (5-25 IU/L), lactate dehydrogenase (LDH) was 2478 U/L (140-280 U/L), and myoglobin was 4329 ng/mL (25-72 ng/mL). He was not relieved from myalgia even after aggressive intravenous fluid resuscitation that prompted rheumatology consultation. He tested positive for 3-hydroxy-3-methylglutaryl-coenzyme-A reductase (HMGCR) IgG antibody, suggesting autoimmune myopathy. He subsequently underwent muscle biopsy, resulting in an inflammatory immune-mediated necrotizing myopathy (IMNM).

It is unclear if HMGCR autoantibodies can directly affect myocytes or if other factors play a role in immune-mediated muscle injury [9]. Anti-signal recognition particles (SRP)+ and anti-HMGCR+ IgG generated from patients harm muscle in vivo via a complement-mediated mechanism that was detected in the sarcolemma of muscle fibers in affected patients. This confirms the autoimmune nature of immune-mediated necrotizing myopathy. These findings support the use of plasma exchanges and argue that complement-targeting therapy should be investigated in IMNM [6].

Patients with SIANM have significant symmetric proximal muscular weakness (95.6%), myopathic electromyography (EMG) findings (97.3%), markedly elevated CPK levels (9718 +/- 7383 IU/L), with positive anti-HMGCR autoantibodies measured by enzyme linked immunosorbent assay (ELISA). Muscle biopsy in SIANM reveals significant degenerating, regenerating, and/or necrotic fibers (100%) [5]. A review of eight cases of SIANM by Needham et al. showed myofiber necrosis and diffuse up-regulation of major histocompatibility complex (MHC)-I expression even in non-necrotic fibers in all cases [10]. Non-necrotic fibers may display higher sarcolemmal staining, and membrane attack complex immunostaining (e.g., C5b-9) may stain the sarcolemma and endothelial cells with varying intensities [11].

Discontinuation of statins and avoidance in the future are the two most critical stages in treating SIANM patients. Most patients improve after starting steroids, immunosuppressants, and, in extreme cases, intravenous immunoglobulin therapy. In a review of eight cases of SIANM by Needham et al., seven improved gradually after starting prednisone and methotrexate, while one improved spontaneously [10].

Our patient's symptoms improved minimally after discontinuing statins and administering oral prednisone 60 mg once daily. He was also advised to avoid statins in the future. Alternative treatment options such as IVIG, methotrexate, and rituximab were also considered. However, the patient declined IVIG due to his cultural beliefs, and methotrexate was not an option for him due to his transaminitis. He was discharged to a rehabilitation facility on oral prednisone 20 mg thrice daily. Rituximab was also considered, and he was asked to follow up with the rheumatologist regarding the same. The patient will be monitored on a long-term basis to comment on the effectiveness of the current treatment plan.

Anti-HMGCR autoantibodies might remain elevated even when the statin is discontinued (given a median statin exposure of 38 months) [12] as chronically elevated HMGCR expression is associated with muscle regeneration [5]. The initial amount of anti-HMGCR antibody measured by ELISA correlates with the blood CPK level and clinical severity at the time of presentation. Decreasing antibody levels is linked to clinical improvement; therefore, it can be used to monitor therapy effectiveness [12]. Patient-reported functional changes and semi-quantitative muscle strength testing or quantitative dynamometry, if available, should be included in the therapeutic monitoring of patients with SIANM. Changes in serum CPK levels can also be utilized as a surrogate biomarker for disease activity, although CPK levels may be persistently elevated even after discontinuing statins [3].

## Conclusions

Health care providers should be aware that anti-3-hydroxy-3-methylglutaryl-coenzyme A reductase (HMGCR) antibodies autoimmune myopathy can arise even after several years of statin use. Anti-HMGCR antibodies and serum CPK remain elevated even after the discontinuation of statins. Further studies are required to determine if we should treat these patients more aggressively to lower antibodies and CPK levels.
